# Supplementary material for: Slotting blasting model experiment and PCA-PNN evaluation model of influencing factors of slitting effect
Source: PLoS One. 2025 Sep 10;20(9):e0330548. doi: 10.1371/journal.pone.0330548 (PMC12422521; doi:10.1371/journal.pone.0330548)
Supplement: Attachment — Submitted filename: R Comments.docx [file pone.0330548.s001.docx]

Based on a thorough review of the manuscript titled "Slotting blasting model experiment and PCA-PNN evaluation model of influencing factors of slitting effect", recommendation for publication:

Peer Review Comments

1. Scientific Contribution and Novelty
   The manuscript presents a compelling integration of experimental blasting analysis with a PCA-PNN hybrid model to evaluate the slotting performance of slotted cartridges. This dual approach offers practical value in both experimental and predictive aspects of blasting engineering. The introduction of PCA for dimensionality reduction before PNN modeling significantly enhances prediction accuracy and model interpretability, showing a meaningful contribution to rock blasting and engineering mechanics.
2. Methodological Rigor and Clarity
   The experimental design is systematic, covering a diverse range of parameters (decoupling coefficient, slotting width, material, etc.) with adequate sample size (28 groups). The use of PMMA as a simulation medium is appropriate, and the detailed measurement of performance indicators (e.g., crack length, damage variable, fractal dimension) demonstrates strong methodological rigor. However, the paper would benefit from additional clarity on how thresholds for classification into G1-G4 performance levels were determined—currently, the rationale appears somewhat empirical.
3. Use of PCA and PNN
   The application of PCA for dimensionality reduction and feature importance analysis is well-justified. The derivation of comprehensive evaluation indicators (F1, F2, F3) is mathematically sound. The PNN implementation is also robust, with spread optimization and multiple training/testing configurations to validate generalization. Nevertheless, some readers may benefit from a more intuitive explanation of why PNN was chosen over other classifiers (e.g., SVM, Random Forest).
4. Figures, Tables, and Interpretation
   Figures and tables are extensive and support the narrative well, especially in visualizing the performance of different parameter sets. The PCA scatter plots, eigenvalue scree plots, and comparative prediction charts clearly show the efficacy of the PCA-PNN model. However, figures could benefit from enhanced labeling (e.g., quadrant indicators or performance-level color coding) to aid reader comprehension.
5. Language and Presentation
   The manuscript is generally readable and technically sound, but the English language usage needs moderate revision for fluency. Some sentences are overly long or have awkward constructions, especially in the introduction and discussion sections. A thorough professional language edit would improve overall readability and impact.
6. Suggestion for Revising References

It is recommended that the author revise the references to incorporate more recent studies in the field. This will ensure the paper reflects the current advancements in slotted cartridge blasting, dimensionality reduction (PCA), and machine learning models like Probabilistic Neural Networks (PNN). Additionally, it would be beneficial to add a comparison with the most recent studies, if available, to highlight how the proposed approach aligns with or advances current research.

Specifically, the author should:

Update references on slotted cartridge blasting to include recent findings on experimental setups, parameter optimization, and performance evaluation, such as the work by Wang et al. (2023) and Yue et al. (2019), which delve into the impact of material, charge structure, and decoupling coefficient on blasting performance.

Revise references related to Principal Component Analysis (PCA) to reflect the latest research, such as Shen et al. (2020) and Zhang et al. (2021), who have explored PCA's role in dimensionality reduction and data simplification for blasting and rock mechanics.

Include comparisons with recent studies on PNN for slotting performance evaluation, such as Xie et al. (2020), who have applied PNN in similar predictive modeling tasks for rock blasting. This comparison highlights the robustness of the PCA-PNN hybrid model presented in the paper.

By updating the references and adding these comparisons, the paper will be more relevant to the current state of the field, demonstrating how the proposed methods build on or improve upon existing models and experimental frameworks.

Recommendation: Accept with Minor Revisions

The manuscript is well-structured and presents original research with high relevance to engineering blasting applications. The hybrid PCA-PNN approach is effective and validated rigorously. Therefore, I recommend acceptance after minor revisions, primarily for language clarity , Revising References and enhanced explanation of classification thresholds and model selection rationale.
